# Supplementary material for: Malnutrition and its determinants among older adults living in foster families in Guadeloupe (French West Indies). A cross-sectional study
Source: PLoS One. 2024 Jun 21;19(6):e0304998. doi: 10.1371/journal.pone.0304998 (PMC11192354; doi:10.1371/journal.pone.0304998)
Supplement: S1 Checklist — (DOCX) [file pone.0304998.s001.docx]

**Human Participants Research Checklist**

***Complete the following if your study involved human participants or human participants’ data. These questions should be addressed for prospective and retrospective studies.***

1. Did you obtain ethics approval for this study?
   - If yes, please upload (file type “Other”) the original approval document you received from your ethics committee. If the original document is in another language, please also provide an English translation.

Yes, but we do not have an English version of our original approval document (in French). You will find attached the favourable opinion of the Ethics Committee 2020-A00620-39.

- - If you did not obtain ethical approval, please explain why this was not required below.

1. If you prospectively recruited human participants for the study – for example, you conducted a clinical trial, distributed questionnaires, or obtained tissues, data or samples for the purposes of this study, please report in the Methods:
   1. the day, month and year of the **start and end** of the recruitment period for this study.
   2. Recruitment opened the 16^th^ November 2020 and ended the 16^th^ April 2021
   3. whether participants provided informed consent, and if so, what type was obtained (for instance, written or verbal, and if verbal, how it was documented and witnessed). If your study included minors, state whether you obtained consent from parents or guardians. If the need for consent was waived by the ethics committee, please include this information.

According to the French legislation, informed consent was obtained. Non-interventional research without any blood or biological samples taken as part of the care (called type 3 researches according to the Jardé law) requires the opinion of an ethics committee and information (oral or written) of the participants. Written consent is not required. An information note has been sent to the participants or their legal representatives, detailing the study and the patients' rights, particularly in terms of personal data protection. Patients have the right to refuse to participate in the study.

1. If you are reporting a retrospective study of medical records or archived samples, please report in the Methods section:
2. the day, month and year when the data were accessed for research purposes
3. whether authors had access to information that could identify individual participants during or after data collection

N/A
